# NH_4_^+^-N alleviates iron deficiency in rice seedlings under calcareous conditions

**DOI:** 10.1038/s41598-019-49207-9

**Published:** 2019-09-03

**Authors:** Xinjiang Zhang, Hui Liu, Shujie Zhang, Juan Wang, Changzhou Wei

**Affiliations:** 1 Key Lab of Oasis Ecology Agriculture of Xinjiang Production and Construction Group, Shihezi University, North 4th Street No. 221, Shihezi, 832000 P.R. China; 2 Special Plant Genomics Laboratory, College of Life Sciences, Shihezi University, North 4th Street No. 221, Shihezi, 832000 P.R. China; 3 Xinjiang Academy of Agriculture and Reclamation, Wuyi Road No. 221, Shihezi, 832000 P.R. China

**Keywords:** Plant physiology, Drought

## Abstract

Drip-irrigated rice (*Oryza sativa* L.) in calcareous soil exhibits signs of iron (Fe) deficiency. This study aimed to explore whether NH_4_^+^ alleviates Fe deficiency in rice seedlings grown under calcareous conditions. Two rice varieties (cv. ‘T43’ Fe deficiency-tolerant variety and cv. ‘T04’ Fe deficiency-sensitive variety) were used to carry out two independent experiments with exposure to different nitrogen (N) forms (nitrate (NO_3_^−^) or NH_4_^+^) under calcareous conditions. In experiment 1, plants were precultured in a nutrient solution with excess Fe (40 µM Fe(II)-EDTA) for 14 d and then supplied NO_3_^−^-N (AN) or NH_4_^−^-N (NN) without Fe for 3, 6, or 12 d. In experiment 2, plants were fed AN or NN with 10 µM Fe(II)-EDTA for 18 d. Compared to plants exposed to AN, leaves of plants exposed to NN showed severe chlorosis and significantly decreased chlorophyll content during Fe starvation. The xylem sap pH and cell wall Fe fraction in both shoots and roots of rice fed NN were significantly higher than those fed AN. However, the Fe concentration in xylem sap, soluble and organelle Fe fractions in both shoots and roots, and the shoot/root Fe content ratio in rice exposed to AN were significantly higher than those in plants exposed to NN. AN reduced the root aerenchyma fraction and root porosity compared to NN, which induced greater water uptake and hydraulic conductance by roots, hence the stronger xylem sap flow rate with AN. The results indicated that NH_4_^+^-N alleviated Fe deficiency in rice under calcareous conditions by promoting Fe re-allocation in rice tissues and Fe transportation from roots to shoots.

## Introduction

Drip-irrigated rice (*Oryza sativa* L.) is a new type of water-saving rice cultivation technology that combines water conservation and high yields^1,2^. However, drip-irrigated rice often suffers from Fe deficiency chlorosis in the calcareous soils of Xinjiang, China^3,4^, whereas flooded rice planted in the same geographical region is not Fe deficient. Therefore, Fe deficiency is an obstacle in the conversion of rice cultivation from flooding irrigation to drip irrigation in calcareous soils^4^.

N, P, K, and Mg are mainly carried from roots to shoots through phloem; however, B, Ca, and Fe are mainly carried to shoots with water in xylem with the help of root pressure and transpiration steam^5,6^. Increased transpiration can promote the transport of Fe from roots to shoots in rice^7^. However, in one study, when rice cultivation was changed from flooding irrigation to drip irrigation, the rate of transpiration from rice decreased significantly^2^. Zhang *et al*.^8^ showed that rice suffers mild drought stress in drip-irrigated soils. Previous studies have indicated that the internal structure of roots influences nutrient uptake^9,10^. In particular, NO_3_^−^-N, higher pH, and well-aerated conditions can cause aerenchyma formation in the roots of rice, which affects its N and water uptake^11–13^. These factors are characteristic of drip-irrigated environments in calcareous soils.

The inorganic N in the flooded soil is mainly found in NH_4_^+^ and that in the upland is mainly found in NO_3_^−^ because of nitrification in aerobic soils^14,15^. Rice is a typical crop that prefers NH_4_^+^ ^16^; the N form in dryland soils is adversely affect N uptake of rice. The assimilation of these two N forms differs significantly in several rhizosphere properties and plant apoplastic pH, which affects the uptake and utilization of Fe. For example, the addition of NO_3_^−^-N increases the apoplastic pH in leaves and decreases the translocation of Fe from apoplasts into cells in sunflowers and maize, thus inducing chlorosis^17,18^. Conversely, plant-assimilated NH_4_^+^-N may lead to acidification of the rhizosphere and apoplasts, which in turn increases Fe absorption by plants^19–21^. Therefore, pH plays an important role in soil Fe availability and plant Fe absorption and transportation.

Based on this understanding, we hypothesized that NH_4_^+^ alleviates Fe nutrition disorders in drip-irrigated rice by (1) promoting Fe re-allocation in rice tissues and (2) inhibiting aerenchyma formation, which increases water and Fe uptake. In the present study, we examined the impact of N forms on Fe uptake, re-allocation, and transport in two different Fe deficiency-tolerant rice varieties grown in simulated drip-irrigated calcareous conditions. Changes in xylem sap pH, Fe concentration in the subcellular fraction of shoots and roots, root aerenchyma formation and porosity, and Fe absorption in rice plants were studied.

## Results

### NH_4_^+^ reduces Fe deficiency chlorosis of rice compared to that with NO_3_^−^

**Figure 1:**
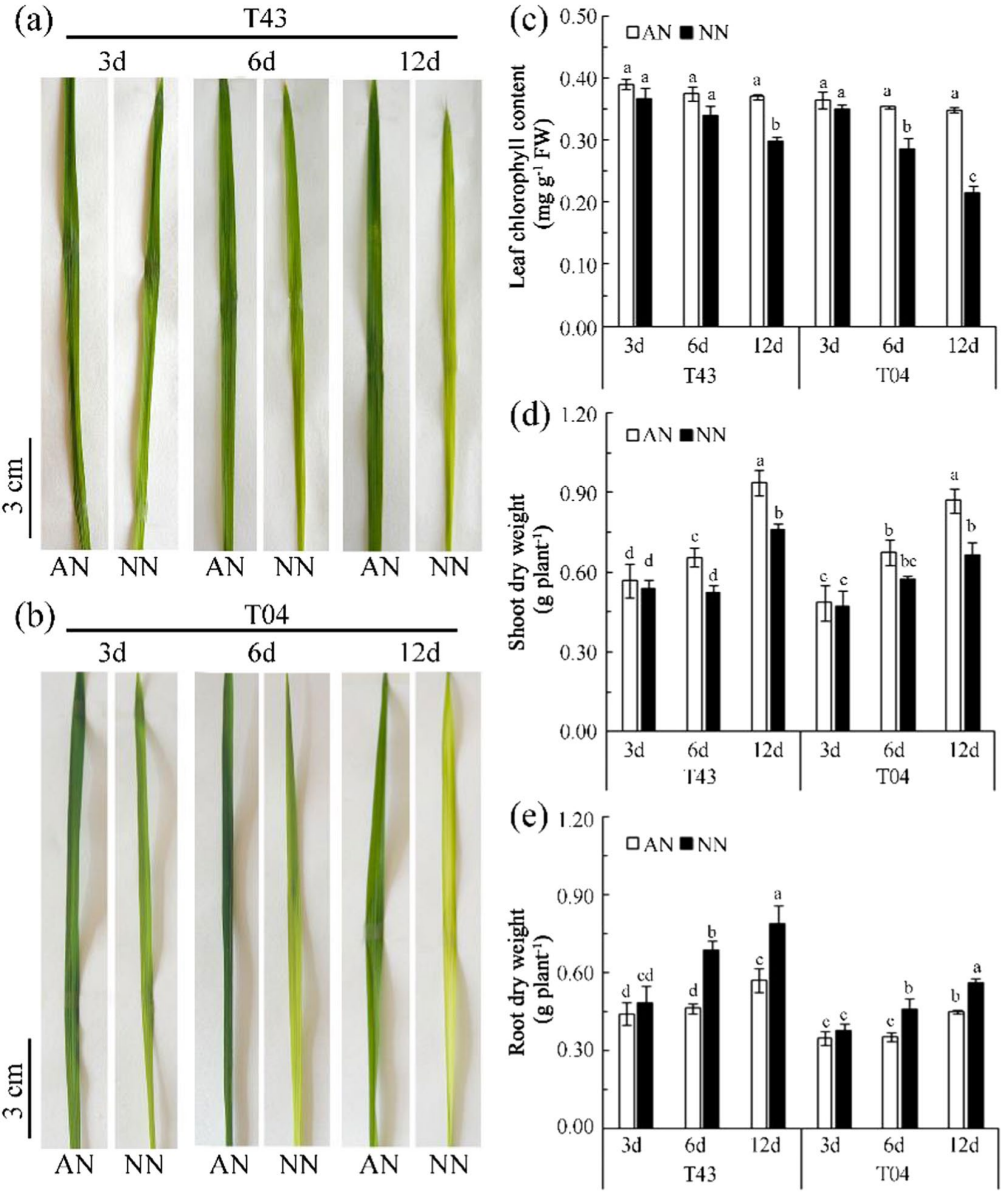
Phenotypes of the first fully expend leaf (**a**,**b**), leaf chlorophyll content in shoots (**c**) and biomass of rice (**d**,**e**) growing in solutions supplied with NH_4_^+^-N (AN) or NO_3_^−^-N (NN) as the N source and buffered at high pH (7.5) during Fe-deficient for 3, 6 and 12 days (‘Experiment 1’). Error bars represent SE (n = 3). The bar graph line within each cultivar the same letter are not significantly different at 5% according to Duncan’s multiple range tests.

**Figure 2:**
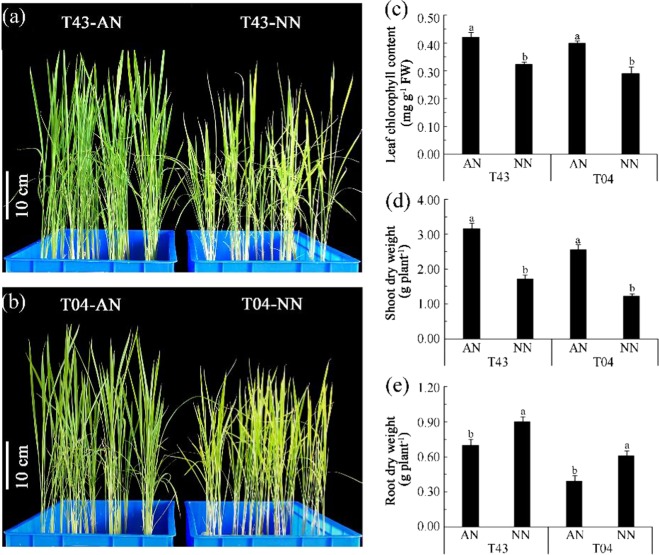
Phenotypes of the total plant (**a**,**b**), leaf chlorophyll content in shoots (**c**) and biomass (**d**,**e**) of rice growing in solutions supplied with NH_4_^+^-N (AN) or NO_3_^−^-N (NN) as the N source and buffered at high pH (7.5) during low EDTA-Fe(II) supplied for 18 days (‘Experiment 2’). Error bars represent SE (n = 3). The bar graph line within each cultivar the same letter are not significantly different at 5% according to Duncan’s multiple range tests.

As shown in Fig. 1 in Experiment 1, there were no significant differences in leaf phenotypes of the two rice varieties (T43 and T04) treated with AN or NN on the 3^rd^ day of Fe starvation (−Fe). However, the leaf phenotypes of these two rice varieties showed visible chlorosis on the 12^th^ day of −Fe with NN treatment (Fig. 1a,b). The leaves chlorophyll content of T43 was not significantly different on the 3^rd^ and 6^th^ days of −Fe with either AN or NN treatment, but it was significantly lower with NN treatment than AN treatment on the 12^th^ day of −Fe (Fig. 1c). The leaves chlorophyll content of T04 showed a significant decrease with NN but not the AN treatment on the 6^th^ and 12^th^ of −Fe (Fig. 1c). As shown in Fig. 2, Experiment 2, after 18 days of low Fe culture in rice, the leaf phenotypes of both rice varieties showed obvious chlorosis with NN treatment (Fig. 2a,b), and the leaves chlorophyll content of both T43 and T04 with NN treatment was significantly lower than that with AN (Fig. 2c).

As shown in Fig. 1d, Experiment 1, the shoot dry weight of T43 with AN treatment was significantly greater than that with NN on the 6^th^ and the 12^th^ day of −Fe, whereas the shoot dry weight of T04 with AN treatment was significantly greater than that with NN only on the 12^th^ day of −Fe. The root dry weight of the two rice varieties following AN treatment was significantly less than that following NN at the 6^th^ and the 12^th^ days of −Fe (Fig. 1e). As shown in Fig. 2, Experiment 2, after 18 days of low-Fe culture, the shoot dry weight of both rice varieties after AN treatment was significantly greater than that after NN (Fig. 2d), whereas the root dry weight with AN was significantly less than that with NN treatment (Fig. 2e).

### NH_4_^+^ improves Fe re-allocation in rice compared to that with NO_3_^−^

**Figure 3:**
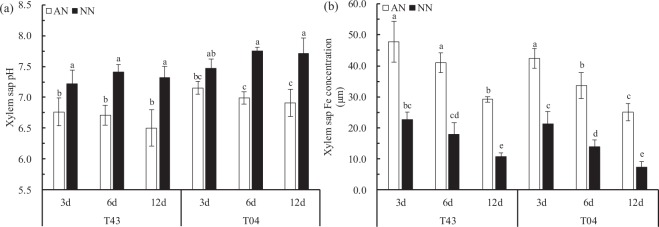
Xylem sap pH (**a**) and xylem sap Fe concentration (**b**) of rice growing in solutions supplied with NH_4_^+^-N (AN) or NO_3_^−^-N (NN) as the N source and buffered at high pH (7.5) during Fe-deficient for 3, 6 and 12 days (‘Experiment 1’). Error bars represent SE (n = 3). The bar graph line within each cultivar the same letter are not significantly different at 5% according to Duncan’s multiple range tests.

As shown in Fig. 3, Experiment 1, the xylem sap pH of both rice varieties (T43 and T04) following AN was significantly lower than that following NN (Fig. 3a). The xylem sap Fe concentration of both rice varieties with AN were significantly higher than that with NN treatment (Fig. 3b).

**Figure 4:**
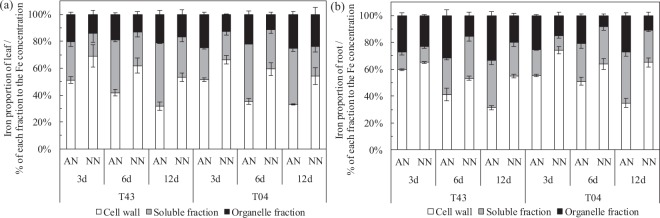
Subcellular distribution proportion of Fe in leaf (**a**) and root (**b**) of rice growing in solutions supplied with NH_4_^+^-N (AN) or NO_3_^−^-N (NN) as the N source and buffered at high pH (7.5) during Fe-deficient for 3, 6 and 12 days (‘Experiment 1’). Error bars represent SE (n = 3).

**Figure 5:**
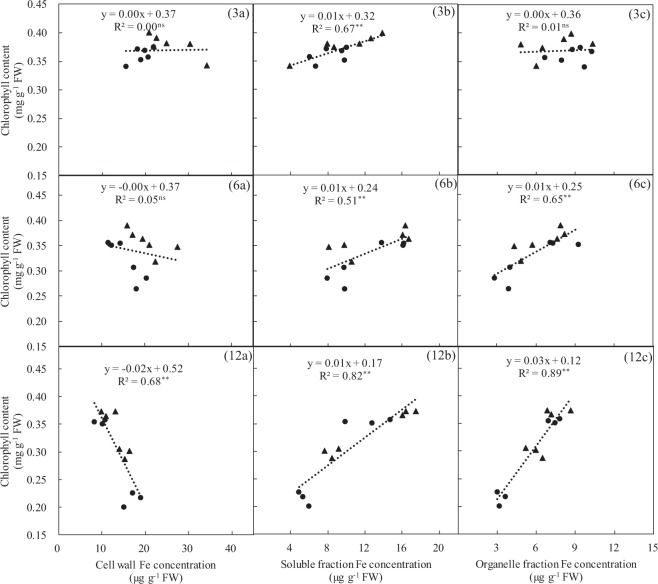
Relationship between leaf subcellular Fe concentration and leaf chlorophyll concentration of rice growing in solutions supplied with NH_4_^+^-N (AN) or NO_3_^−^-N (NN) as the N source and buffered at high pH (7.5) during Fe-deficient for 3 (**3a**, **3b** and **3c**), 6 (**6a**, **6b** and **6c**) and 12 days (**12a**, **12b** and **12c**) (‘Experiment 1’). Filled triangles and filled circles represent T43 and T04, respectively. Lines represent linear regressions. “**” or “ns”: significant at p < 0.01 or p > 0.5.

As shown in Figs 4 and 5, Experiment 1, the proportion of subcellular Fe distributed in the leaves and roots of these two rice varieties were as follows: the cell wall Fe fraction following AN was significantly less than that following NN treatment, whereas the soluble and organelle Fe fractions after AN were significantly higher than that after NN treatment. With an increased number of −Fe days, the cell wall Fe fraction in the leaves and roots of these two rice varieties gradually decreased, and the proportion of corresponding soluble and organelle Fe components gradually increased (Fig. 4a,b). In addition, with the prolongation of −Fe time, the chlorophyll content in rice leaves showed a negative correlation with the Fe concentration in cell wall components (Fig. 5 (3a, 6a, and 12a)), and the chlorophyll content of rice leaves showed a positive correlation with Fe concentration in the organelle fraction (Fig. 5 (3c, 6c, and 12c)).

As shown in Table 1, Experiment 1, on the 3^rd^ day of −Fe, there was no significant difference in Fe concentration and Fe content in shoots and roots of either of these two rice varieties after AN and NN treatment. On the 6^th^ and 12^th^ days of −Fe, both the Fe concentration and Fe content in shoots of these two rice varieties were significantly higher following AN versus NN treatment; however, the Fe concentration and Fe content in roots of these two rice varieties were significantly lower after AN than NN treatment. Correspondingly, the shoot/root Fe ratio content of T43 following AN was always significantly higher than that following NN throughout the whole −Fe period. There was no significant difference in the shoot/root ratio of Fe content in T04 between the AN and NN treatments on the 3^rd^ day of −Fe, whereas the shoot/root ratio of Fe content in T04 with AN treatment was significantly higher than that with NN treatment at the 6^th^ and 12^th^ days of −Fe (Table 1).

**Table 1:** Fe concentration, Fe content and shoot/root Fe content ratio of rice growing in solutions supplied with NH_4_^+^-N (AN) or NO_3_^−^-N (NN) as the N source and buffered at high pH (7.5) during Fe-starvation for 3, 6 and 12 days (‘Experiment 1’).

Rice varieties	Fe deficiency days (d)	N forms	Fe concentration (µg g^−1^)		Fe content (µg plant^−1^)		Fe content ratio (%)
			Shoot	Root	Shoot	Root	Shoot/root
T43	3 d	AN	229 ± 16a	453 ± 46a	130 ± 21ab	199 ± 24b	66 ± 14c
		NN	208 ± 14ab	497 ± 17a	112 ± 14b	241 ± 27ab	47 ± 5d
	6 d	AN	193 ± 6b	302 ± 17c	126 ± 10ab	140 ± 7c	91 ± 7b
		NN	146 ± 11c	376 ± 36b	76 ± 5c	258 ± 20a	30 ± 4d
	12 d	AN	163 ± 10c	230 ± 29d	152 ± 4a	131 ± 17c	118 ± 15a
		NN	109 ± 15d	304 ± 24c	83 ± 12c	241 ± 36ab	35 ± 0d
T04	3 d	AN	185 ± 7a	611 ± 76ab	90 ± 14ab	213 ± 44b	43 ± 7b
		NN	173 ± 9ab	689 ± 77a	83 ± 14bc	258 ± 15a	32 ± 6bc
	6 d	AN	165 ± 9b	481 ± 19c	111 ± 8a	169 ± 3c	66 ± 4a
		NN	131 ± 4c	528 ± 14b	75 ± 3bc	242 ± 20ab	31 ± 2c
	12 d	AN	125 ± 5c	348 ± 17d	109 ± 9a	156 ± 7c	70 ± 7a
		NN	97 ± 11d	463 ± 19c	65 ± 12c	260 ± 10a	25 ± 4c

Values within a column and within each cultivar, the same letter are not significantly different at 5% by Duncan’s multiple range tests.

### NH_4_^+^ improves xylem sap fluid rate and xylem sap Fe concentration of rice compared to those with NO_3_^−^

As shown in Table 2, Experiment 2, both the xylem sap flow rate and xylem sap Fe concentration in these two rice varieties (T43 and T04) with AN treatment were significantly higher than those treated with NN under a low Fe supply.

**Table 2:** Xylem sap flow rate and xylem sap Fe concentration of rice growing in solutions supplied with NH_4_^+^-N (AN) or NO_3_^−^-N (NN) as the N source and buffered at high pH (7.5) during low EDTA-Fe(II) supplied for 18 days (‘Experiment 2’).

Rice varieties	N forms	Xylem sap flow rate (mg h^−1^ plant^−1^)	Xylem sap Fe concentration (µm)
T43	AN	50.36 ± 5.61a	13.33 ± 1.64a
	NN	29.29 ± 5.87b	7.47 ± 0.52b
T04	AN	46.14 ± 6.21a	7.65 ± 0.27a
	NN	23.25 ± 3.89b	5.43 ± 0.45b

Values within a column and within each cultivar the same letter are not significantly different at 5% by Duncan’s multiple range tests.

### NH_4_^+^ enhances Fe transport from roots to shoots of rice compared to that with NO_3_^−^

**Figure 6:**
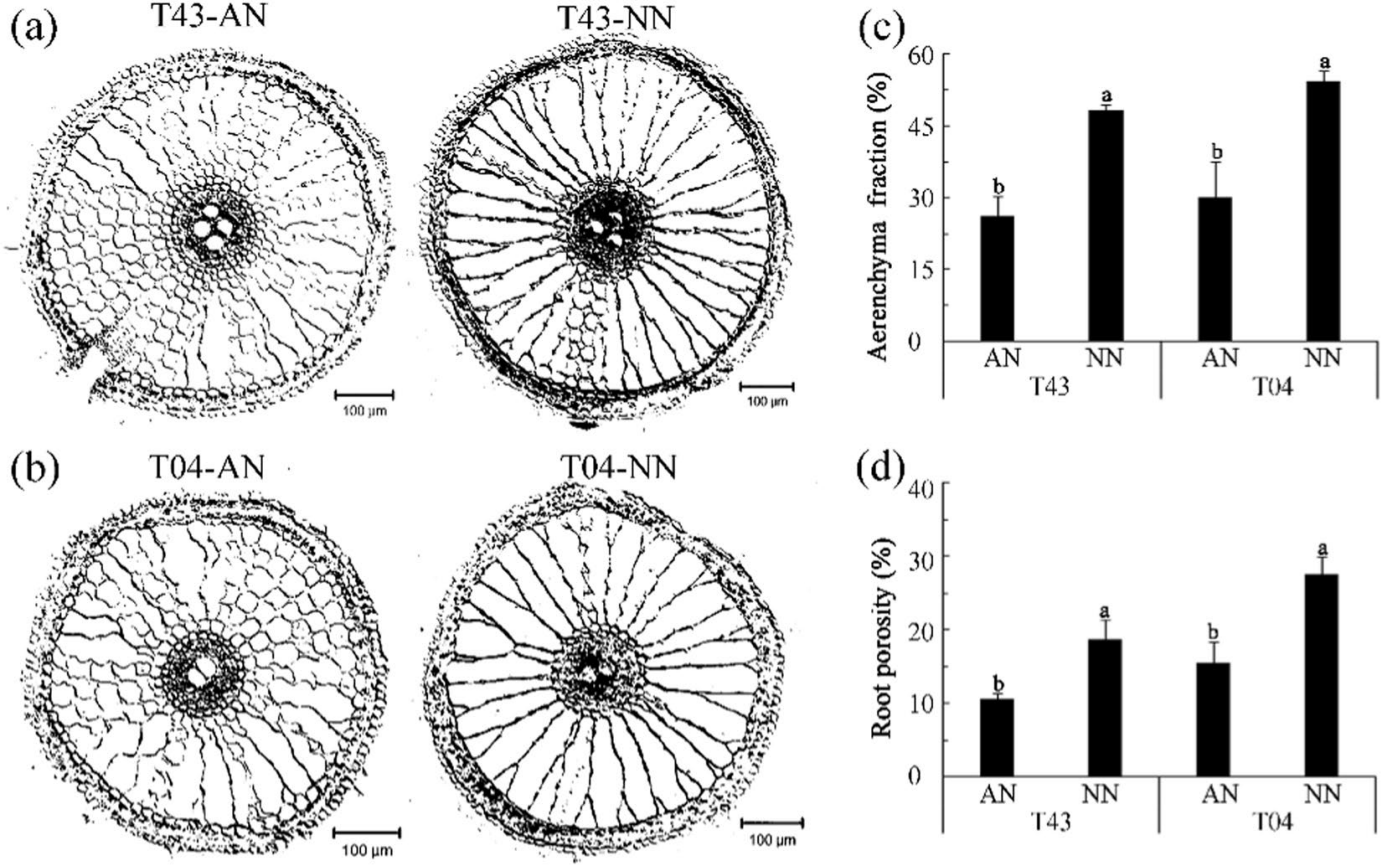
Cross-sections of the root tip (2 cm (**a**,**b**)), root aerenchyma fraction (**c**) and root porosity (**d**) of rice growing in solutions supplied with NH_4_^+^-N (AN) or NO_3_^−^-N (NN) as the N source and buffered at high pH (7.5) during low EDTA-Fe(II) supplied for 18 days (‘Experiment 2’). Error bars represent SE (n = 3). The bar graph line within each cultivar the same letter are not significantly different at 5% according to Duncan’s multiple range tests.

**Figure 7:**
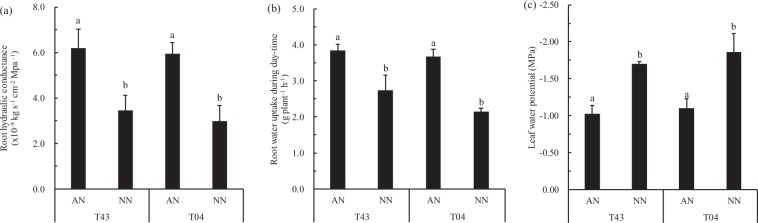
Root hydraulic conductance (**a**), root water uptake rates during daytime (**b**) and leaf water potential (**c**) of rice growing in solutions supplied with NH_4_^+^-N (AN) or NO_3_^−^-N (NN) as the N source and buffered at high pH (7.5) during low EDTA-Fe(II) supplied for 18 days (‘Experiment 2’). Error bars represent SE (n = 3). The bar graph line within each cultivar the same letter are not significantly different at 5% according to Duncan’s multiple range tests.

**Figure 8:**
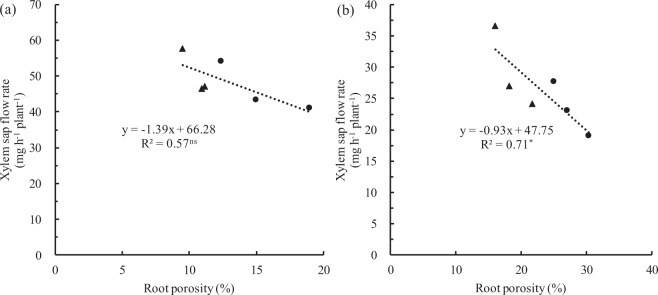
Relationship between root porosity and xylem sap flow rate of rice growing in solutions supplied with NH_4_^+^-N (AN (**a**)) or NO_3_^−^-N (NN (**b**)) as the N source and buffered at high pH (7.5) during low EDTA-Fe supplied for 18 days (‘Experiment 2’). Filled triangles and filled circles represent T43 and T04, respectively. Lines represent linear regressions. “*” or “ns”: significant at p < 0.05 or p > 0.5.

As shown in Experiment 2, the root aerenchyma fraction and root porosity of these two rice varieties (T43 and T04) were significantly lower following AN versus NN treatment (Fig. 6). The root hydraulic conductance, root water uptake, and leaf water potential of these two rice varieties was significantly higher with AN compared to NN treatment (Fig. 7). A strong negative correlation was observed between the xylem sap flow rate and root porosity with NN treatment (Fig. 8b), whereas the correlation was weak with AN treatment (Fig. 8a). The Fe concentration and Fe content in shoots and shoot/root ratio of Fe content in these two rice varieties (T43 and T04) following AN treatment were significantly higher than those following NN treatment, whereas the Fe concentration and Fe contents in roots of these two rice varieties (T43 and T04) after AN treatment were significantly lower than those in the NN treatments (Table 3).

**Table 3:** Fe concentration, Fe content and shoot/root Fe content ratio of rice growing in solutions supplied with NH_4_^+^-N (AN) or NO_3_^−^-N (NN) as the N source and buffered at high pH (7.5) during low EDTA-Fe(II) supplied for 18 days (‘Experiment 2’).

Rice varieties	N forms	Fe concentration (µg g^−1^)		Fe content (µg plant^−1^)		Fe content ratio
		Shoot	Root	Shoot	Root	(%) Shoot/root
T43	AN	183 ± 14a	392 ± 26b	580 ± 19a	273 ± 13b	213 ± 10a
	NN	103 ± 23b	745 ± 58a	175 ± 31b	671 ± 61a	27 ± 7b
T04	AN	143 ± 27a	436 ± 26b	258 ± 59a	172 ± 27b	154 ± 44a
	NN	77 ± 12b	901 ± 34a	93 ± 13b	550 ± 56a	17 ± 4b

Values within a column and within each cultivar the same letter are not significantly different at 5% by Duncan’s multiple range tests.

## Discussion

Previous studies have shown that most Fe in leaf apoplasts (>95%) is bound to cell walls^22^, and reutilizing this store of Fe is a key strategy used by plants to avoid Fe deficiency chlorosis at nutrient solutions with a pH of 5.5^23^. In the present study, we examined whether different N forms exhibit mobilization effects on cell wall Fe in rice under calcareous conditions.

In rice seedlings under Fe starvation for 12 days and at pH 7.5, the proportion of Fe in cell walls in both roots and leaves of these two rice varieties was significantly less with AN than NN treatment, and the solution fraction and organelle fraction of Fe gradually increased (Fig. 4). This means that NH_4_^+^ may mobilize a portion of the Fe that is bound to cell walls to enter the cell solution and organelles under Fe-deficient conditions. To test this conjecture, we fitted a linear relationship between the Fe concentration of different subcellular components and chlorophyll content of leaves from the two rice varieties. With prolonged Fe starvation, leaf cell wall Fe and chlorophyll content showed a strong and significant negative correlation (Fig. 5 (3a, 6a, and 12a)), and organelle Fe showed a strong and significant positive correlation with chlorophyll content (Fig. 5 (3c, 6c, and 12c)). These results explain why the chlorophyll content of rice leaves showed no significant changes with AN treatments (Fig. 1a–c). The reason that cell wall Fe was significantly greater with NN than AN treatment was xylem alkalization (Fig. 3). Similar results were reported by Kosegarten *et al*.^17^. They found that the xylem sap pH in the leaves of sunflowers was higher with alkaline nutrition (NO_3_^−^ and NO_3_^−^/HCO_3_^−^) treatment versus NH_4_NO_3_ treatment. The activity of Fe(III) reductase in roots and leaves depends on the pH of apoplasts, and apoplast pH directly affects the reduction and absorption of Fe in plant tissues^24–26^. The optimum pH range for Fe(III) reductase in leaves is 5.0–6.8^27^. In this study, although the rice was grown in solution with a pH of 7.5 buffered by HCO_3_^−^ and N-(2-hydroxyethyl)-piperazine-N′-2-ethanesulfonic acid (HEPES), there was still a big difference in the xylem sap pH of rice treated with different N forms. For example, the pH of xylem sap fluid in each rice variety with AN treatment was within the optimum pH range for Fe(III) reductase activity, with means of approximately 6.66 (T43) and 7.02 (T04), respectively, whereas the mean pH values for xylem sap fluid in each rice variety with NN treatment were approximately 7.32 (T43) and 7.64 (T04), respectively (Fig. 3a). Compared with NN treatment, the xylem sap fluid pH with AN treatment was more suitable for Fe(III) reductase to exert its physiological function of reducing Fe in the cell walls under alkaline conditions, which should increase the proportion of Fe distributed in the cell solution and cell organelles (Fig. 4). Furthermore, Kosegarten *et al*.^17^ indicated that when the pH in xylem apoplasts from sunflowers increased from 6.86 to 7.71, the Fe(III) reductase activity of sunflower leaves decreased rapidly from 54% to 22%. Consequently, we draw the conclusion that when rice suffers from Fe starvation in calcareous conditions, feeding the rice with NH_4_^+^-N can release Fe stored in cell walls by alleviating the xylem alkalization induced by NO_3_^−^-N to ensure normal metabolic activity in organelles. In addition, the Fe concentration in rice xylem sap and shoot/root Fe content with AN treatment were significantly higher than those with NN treatment (Fig. 3b, Table 1). This means that NH_4_^+^ not only improved the Fe utilization efficiency at the subcellular level in rice but also increased the Fe transport efficiency from roots to shoots, similar to what was reported in maize by Zou *et al*.^28^.

Aerenchyma at the root cortex impeded the radial transport of water in root cylinders and decreased water uptake in water-stressed rice plants fed NO_3_^−^-N^12,29^. Under water stress, both stomatal conductivity and leaf water potential were greater in rice fed NH_4_^+^-N versus those fed NO_3_^−^-N^30,31^. These effects should be expected to enhance the availability of water for shoot growth while favoring transpiration. To further determine the factors affecting the transport of Fe in rice under calcareous conditions, we examined the effects of different N treatments on water absorption and Fe transport from roots to shoots of rice, especially in roots fed NH_4_^+^-N under calcareous conditions. The results showed that NO_3_^−^-N restricted shoot growth (Figs 1d and 2a,b,d) and that aerenchyma and pores formed in roots fed with NO_3_^−^-N (Fig. 6), suggesting that root aerenchyma and root porosity are regulated by N forms, perhaps because NO_3_^−^ promotes ethylene production in rice roots^12^. However, root water uptake capacity, indicated by transpiration, root hydraulic conductance, and leaf water potential in rice treated with AN were significantly greater than those treated with NN (Fig. 7). These results suggest that NH_4_^+^-N can alleviate water stress in rice better than NO_3_^−^-N, as has been reported previously^32,33^. In particular, a significant negative correlation was observed between xylem sap flow rate and root porosity in plants fed NO_3_^−^-N (Fig. 8b), whereas no significant correlation was found in plants fed NH_4_^+^-N (Fig. 8a). Therefore, NH_4_^+^ can improve the water absorption capacity of roots by inhibiting aerenchyma formation and porosity of roots under water stress in calcareous conditions. Moreover, the data from both Experiment 1 and Experiment 2 showed that the leaf chlorophyll content, Fe concentration in xylem sap, and ratio of shoot/root Fe content in these rice varieties following AN treatment were higher than those following NN treatment (Figs 1c, 2c and 3b, Tables 1–3). Collectively, this evidence suggests that NH_4_^+^ can not only improve root water absorption but also increase rice Fe translocation. Because Fe is mainly transferred through transpiration fluid^5,6^ and drip-irrigated rice easily suffers from water stress^2,8^, improved root water absorption capacity can drive more Fe into the xylem for transfer from the roots to the shoots of rice fed NH_4_^+^-N under calcareous conditions.

## Conclusion

Drip irrigation of rice is a water-saving technology; however, Fe deficiency is a side effect of this method because of xylem alkalization and less water uptake by rice fed NO_3_^−^. The application of NH_4_^+^ to rice can reduce Fe deficiency through two mechanisms: (1) release of Fe absorbed in cell walls by alleviating xylem alkalization induced by NO_3_^−^ and maintaining more of the Fe in organelles and cell fluid and ensuring normal physiological activity in rice, (2) absorption of NH_4_^+^ by rice, which can reduce the aerenchyma fraction and porosity of roots, thereby improving the water absorption capacity of roots and promoting transport of more Fe from roots to shoots.

## Materials and Methods

### Growth conditions and plant culture

Two independent experiments were conducted to study the effects of N forms on Fe re-allocation and transport in rice (see below for the experimental design). Simulated drip-irrigated calcareous soil solution conditions were realized by adding 5 mM KHCO_3_ and 5 mM HEPES to the nutrient solution to stabilize its pH at 7.5. Water stress was simulated by adding 3.5% (w/v) polyethylene glycol (PEG, MW 6000) to adjust the water potential at −0.03 MPa, equivalent to 90–95% field water capacity^34^. For aeration conditions, air was pumped continuously into the nutrient solution using an air pump (aquarium air pump AP228, max output: 6 L/min, China).

Before culture, seeds from rice (Fe deficiency-tolerant [*Oryza sativa* L. cv. T43] and Fe deficiency-sensitive [*Oryza sativa* L. cv. T04] varieties^35^) were disinfected in 10% H_2_O_2_ (w/w) for 30 min and then germinated in saturated CaSO_4_ solution for 3 days. After the seedlings developed an average of 2.5 visible leaves in distilled water, they were transplanted into a 6-L plastic box containing a quarter-strength mixture of NO_3_^−^ and NH_4_^+^ nutrient solution (see below for solution compositions) for 7 days. After 7 days of growth, the seedlings were transferred to a half-strength mixture of NO_3_^−^ and NH_4_^+^ nutrient solution, and water stress was simulated by adding 1.5% PEG (6000) to the nutrient solutions. Seven days later, the seedlings were supplied with a full-strength mixture of NO_3_^−^ and NH_4_^+^ in the nutrient solution for further growth, and water stress was simulated by adding 3.5% PEG (6000) to the nutrient solutions for an additional week. Then, the seedlings were used in the following two independent experiments. During pre-culture of rice seedlings, the pH of the nutrient solutions was adjusted to 5.50 ± 0.05 by adding 0.1 M HCl or 0.1 M NaOH every day. After N form treatments (Experiments 1 and 2, see below), the nutrient solutions were buffered with 5 mM HEPES at pH 7.5 and adjusted daily with HCl or NaOH. These culture solutions were renewed every 2 d. Plants were grown in a greenhouse with a light/dark regime of 16/8 h and a temperature of 28/20 °C (day/night). In the 16-h photoperiod, plants received a photon flux density of 500 μmol m^−2^ s^−1^ of photosynthetically active radiation (SON-T AGRO 400 W bulbs) at the leaf level in a relative humidity of 75–85%.

The nutrient solution composition (NSC) for culturel, defined by the International Rice Research Institute, was prepared as described by Guo *et al*.^36^ with minor modifications. Macronutrients (mM) were present as follows: 2.85 N as (NH_4_)_2_SO_4_ or Ca(NO_3_)_2_, 5 K and HCO_3_^−^ as KHCO_3_, 0.32 P as KH_2_PO_4_, and 1.65 Mg as MgSO_4_. Micronutrients (µM) were present as follows: 9.10 Mn as MnSO_4_, 0.15 Zn as ZnSO_4_, 0.16 Cu as CuSO_4_, 18.5 B as H_3_BO_3_, 0.52 Mo as (NH_4_)_6_Mo_7_O_24_, and 0.1 Si as Na_2_SiO_4_, using 5 mM KHCO_3_ as a substitute for NaHCO_3_ to avoid expose of rice to a salt environment. In the NH_4_^+^-only nutrient solution, Ca^2+^ was supplied as CaCl_2_ (1.43 mM). The nitrification inhibitor dicyandiamide (0.01 mM) was added to all pots to keep NH_4_^+^ stable in the solution.

### Experiment 1

Pre-cultured rice seedlings were cultured with 40 µM Fe(II)-EDTA in a full-strength mixture of NO_3_^−^ and NH_4_^+^ for an additional 14 days, and then the seedlings of each cultivar (T43 and T04) were divided into two groups for NH_4_^+^-N (AN) or NO_3_^−^-N (NN) treatment. The AN and NN treatments had nutrient strengths identical to that of the NSC but without Fe(II)-EDTA. Each group (treatment) comprised three boxes (replicates) of rice seedlings, and each box had 24 holes with two rice seedlings per hole. The position of each box was changed every 2 days to avoid edge effects in the greenhouse. After 3, 6, and 12 days of Fe starvation, the plants were harvested and divided into shoots and roots, and leaf chlorophyll contents, xylem sap pH, xylem sap Fe concentrations, and subcellular fractions Fe tissue concentrations were determined.

### Experiment 2

Pre-cultured rice seedlings of each cultivar (T43 and T04) were divided into two groups for AN and NN treatment. Each group (treatment) comprised three boxes (replicates) of rice seedlings, and each box had 24 holes with two rice seedlings per hole. The position of each box was changed every 2 days to avoid edge effects in the greenhouse. The AN and NN treatments had the same N content as that of the NSC with low Fe(II)-EDTA (10 µM) supplied for 18 days. At harvest, leaf chlorophyll content, leaf water potential, xylem sap flow rate, xylem sap Fe concentration, root aerenchyma, root porosity, root water uptake rate, root hydraulic conductance, and Fe concentrations in shoots and roots were analyzed.

### The recorded parameters are described below

#### Xylem sap collection, xylem sap flow rate, xylem sap pH, xylem sap Fe concentration measurements

The day before the collection of other test indicators, three-hole of seedlings were used to collect xylem sap as a data of each replicate (about 2 mL per replicate). Xylem sap was collected following the method of Yang *et al*.^29^. Briefly, plants were de-topped approximately 2 cm above the interface of the roots and shoots, and the exudation was immediately cleaned with filter paper to avoid contamination. Absorbent cotton was placed on the top of each piece of de-topped xylem and covered with plastic film to avoid evaporation. The xylem sap was collected from 21:0 h to 09:00 h, and the xylem sap flow rate was calculated from the differences in cotton weight and collection time. Then, placed the cotton in a centrifuge tube and centrifuge at 4000 × g for 10 min at 4 °C to obtain xylem sap fluid. The xylem sap pH was measured with a combined electrode pH meter (Spectrum Technologies, Inc. IQ 150, USA) immediately. Collected sap samples were later diluted directly in 5% HNO_3_ solution and Fe concentration was determined by an atomic absorption spectrophotometer (Hitachi Z-2000, Japan)^37^.

#### Sample harvest and Fe concentration measurements

Three-hole of seedlings in each treatment which used to collect the xylem sap as described above were harvested and separated into shoots and roots. The shoot samples washed with running tap water followed by acidified solution (0.1 M HCl) and the root samples washed with running tap water followed by 0.5 mM CaSO_4_ solution to remove contaminants, and the samples were then washed several times with deionized water. Then, the samples were dried at 105 °C for 30 min, and at 70 °C for 48 h to constant weight. The biomass (dry weight, DW) was determined. Furthermore, the dried plant tissues were ground with a stainless-steel mill to pass through a 0.5 mm sieve. The triturated plant tissues (about 0.5 g) were digested at 120 °C with an acid oxidative mixture of HNO_3_ and HClO_4_ (4:1) until the solution became transparent^37^. After cooling, Fe concentrations of the extract were determined by an atomic absorption spectrophotometer (Hitachi Z-2000, Japan).

#### Separation and determination of Fe in the subcellular fractions

Three-hole of seedlings in each treatment were used to determine of Fe in the subcellular fractions. Separation and determination of Fe in the subcellular fractions were prepared as described by Liu *et al*.^38^ with minor modifications. Briefly, frozen new fully expanded leaves and root tip tissues (0.5 g) were homogenized using a chilled mortar and a pestle in a pre-cold extraction buffer containing 50 mM Tris–HCl (pH 7.5), 250 mM sucrose and 1.0 mM dithioerythritol. The homogenate was then transported into a 50 mL centrifugal tube and centrifuged at 300 × g for 5 min using a high-speed refrigerated centrifuge (Lynx 6000, Thermo Fisher, Germany). The pellet was considered as cell wall fraction. The supernatant was further centrifuged at 20,000 × g for 45 min to sediment cell organelles. The pellet was taken as organelle fraction. The resultant supernatant solution was referred as soluble fraction. All homogenizations and subsequent fractionations were performed at 4 °C. Each pooled solution was evaporated on an electric plate at 70 °C to constant weight. The fractions were dried and digested at 120 °C with an acid oxidative mixture of HNO_3_ and HClO_4_ (4:1) until the solution became transparent^37^. Fe concentrations in the fractions were determined by an atomic absorption spectrophotometer (Hitachi Z-2000, Japan).

#### Chlorophyll measurements

Two-hole of plants were used to determine chlorophyll. The fresh leaves (about 0.2 g) were weighed and ground into a powder in liquid nitrogen. Chlorophyll was extracted from the tissue by adding 8 mL of 80% (v:v) acetone, followed by incubation in complete darkness for 12 h. After centrifugation at 15,000 × g for 5 min, the extract was analyzed at 663 and 645 nm with a spectrometer (UV-2600, Shimadzu, Japan). The total chlorophyll content (mg g^−1^ fresh weight) in shoots was calculated as described by Li *et al*.^31^.

#### Determination of water uptake rate of root and leaf water potential

Water uptake of intact roots was determined by the depletion of nutrient solution (weighing) between 09:00 and 12:00 as described by Gao *et al*.^39^. Leaf water potential was determined with a WP4C Dewpoint Potential Meter (Decagon Devices, USA). The newly expanded leaves were cut into 0.4 cm length pieces, and water potential was measured immediately.

#### Root aerenchyma and porosity measurements

Approximately 7–8 cm long newly formed adventitious roots were selected for aerenchyma measurements. Root tissue was prepared as described previously^13^ with minor modifications for the use of Technovit-7100 embedding resin (Kulzer, Germany) according to the manufacturer’s specifications. Excised root segments were 2.0–2.5 cm (where aerenchyma development is completed) from the root tip. The sections were observed under a confocal laser scanning microscope (LSM 710 NLO, Zeiss, Germany), and aerenchyma formation was calculated from section images using Image J software. The porosity of the adventitious roots was determined according to previous methods^29^. Briefly, 0.8–1.0 g of fresh adventitious roots were detached with a razor blade, cut into about 1–2 cm length segments, and weighed (*RFW*, root fresh weight). A 25 mL Pyrex pycnometer flask, full of degassed water, was weighed before (*W*_1_) and after (*W*_2_) adding these segments. Then, the root segments were transferred to a scintillation vial, which was also filled with degassed water, and vacuum-infiltrated until no air bubbles were detected. These vacuum-infiltrated root segments were retransferred to the above degassed water-filled flask, and the flask was weighed again before (*W*_3_) and after (*W*_4_) adding these segments. Water temperature was measured after each weighing to correct the weight readings. Root porosity was calculated as follows:

1 $$Porosity( \% )=[(({W}_{4}-{W}_{3})-({W}_{2}-{W}_{1}))/({W}_{1}+RFW-{W}_{2})]\times 100$$

#### Root hydraulic conductivity measurements

Root hydraulic conductance was measured using a high-pressure flow meter (HPFM; Decagon Devices, Pullman, WA, USA). Rice plants were topped approximately 2 cm above the root/shoot interface and the HPFM was attached to the detached root using an omnifit connector. Positive pressure (*Pi*) was applied to force water from the base of the excised root to the root tip (opposite to the normal direction of flow during transpiration). The *Pi* at the base was increased rapidly from 0 to 0.5 MPa at a constant rate of 3–7 kPa s^−1^ while measuring the flow (*F*) and applied pressure (*Pi*) every few seconds. The slope of the relationship between *F* and *Pi* was taken as a transient measurement (*Kr*, kg s^−1^ MPa^−1^). After *Kr* was measured using transient methods, root surface areas (*Sr*) were measured using a flatbed image scanner (Epson Expression/STD 1600 Scanner) and a WinRHIZO 2008a software (Regent Instruments). Root hydraulic conductivity (*LPr*, kg s^−1^ cm^−2^ MPa^−1^) was calculated using the equation:

2 $$L\,{\rm{\Pr }}=Kr/Sr$$

### Statistical analysis

SPSS software Version 18.0 for Windows and Microsoft Excel 2016 were used to perform standard statistical tests via one-way analysis of variance. Significant differences were determined by Duncan’s multiple range test at P < 0.05.
