# Author Correction: NH_4_^+^-N alleviates iron deficiency in rice seedlings under calcareous conditions

**DOI:** 10.1038/s41598-020-69146-0

**Published:** 2020-07-17

**Authors:** Xinjiang Zhang, Hui Liu, Shujie Zhang, Juan Wang, Changzhou Wei

**Affiliations:** 1 Key Lab of Oasis Ecology Agriculture of Xinjiang Production and Construction Group, Shihezi University, North 4th Street No. 221, Shihezi, 832000 P.R. China; 2 Special Plant Genomics Laboratory, College of Life Sciences, Shihezi University, North 4th Street No. 221, Shihezi, 832000 P.R. China; 3 Xinjiang Academy of Agriculture and Reclamation, Wuyi Road No. 221, Shihezi, 832000 P.R. China

Correction to: *Scientific Reports* 10.1038/s41598-019-49207-9, published online 03 September 2019

This Article contained errors.

As a result of a typographic error, in the Abstract the abbreviation for nitride oxide nitrogen and ammonium nitrogen were swapped. Additionally, ammonium ion was incorrectly marked as an anion. Therefore in the Abstract,

“In experiment 1, plants were precultured in a nutrient solution with excess Fe (40 μM Fe(II)-EDTA) for 14 d and then supplied NO3^−^-N (AN) or NH4^−^-N (NN) without Fe for 3, 6, or 12 d."

now reads:

“In experiment 1, plants were precultured in a nutrient solution with excess Fe (40 μM Fe(II)-EDTA) for 14 d and then supplied NO3^−^-N (NN) or NH4^+^-N (AN) without Fe for 3, 6, or 12 d.”

These abbreviations were used correctly elsewhere in the Article; the conclusions are therefore not affected by these changes.

This has now been corrected in the PDF and HTML versions of the Article.

